# Deep learning for Arabic healthcare: MedicalBot

**DOI:** 10.1007/s13278-023-01077-w

**Published:** 2023-04-18

**Authors:** Mohammed Abdelhay, Ammar Mohammed, Hesham A. Hefny

**Affiliations:** Department of Computer Science, Faculty of Graduate Studies for Statistical Research, Cairo University, Giza, Egypt

**Keywords:** Deep learning, LSTM and attention, NLP, Q$\&$A Arabic corpus, Transformers

## Abstract

Since the COVID-19 pandemic, healthcare services, particularly remote and automated healthcare consultations, have gained increased attention. Medical bots, which provide medical advice and support, are becoming increasingly popular. They offer numerous benefits, including 24/7 access to medical counseling, reduced appointment wait times by providing quick answers to common questions or concerns, and cost savings associated with fewer visits or tests required for diagnosis and treatment plans. The success of medical bots depends on the quality of their learning, which in turn depends on the appropriate corpus within the domain of interest. Arabic is one of the most commonly used languages for sharing users’ internet content. However, implementing medical bots in Arabic faces several challenges, including the language’s morphological composition, the diversity of dialects, and the need for an appropriate and large enough corpus in the medical domain. To address this gap, this paper introduces the largest Arabic Healthcare Q &A dataset, called MAQA, consisting of over 430,000 questions distributed across 20 medical specializations. Furthermore, this paper adopts three deep learning models, namely LSTM, Bi-LSTM, and Transformers, for experimenting and benchmarking the proposed corpus MAQA. The experimental results demonstrate that the recent Transformer model outperforms the traditional deep learning models, achieving an average cosine similarity of 80.81% and a BLeU score of 58%.

## Introduction

The main problem with developing Arabic language bots is the lack of task-oriented dialogue datasets, which causes Arabic to lag in the conversational artificial intelligence (AI) systems development (AlHagbani and Khan 2016). Additionally, Arabic has rich morphology, orthographic variations, ambiguity, and multiple dialects, which make it a more challenging language for bot development (Alhassan et al. 2022). The technology for the Arabic language is still in its infancy stage, and several obstacles and challenges need to be resolved to develop an effective Arabic bot (AlHagbani and Khan 2016). Therefore, the lack of task-oriented dialogue datasets and the complexity of the Arabic language poses significant challenges to developing successful Arabic language bots (Alhassan et al. 2022).

The main contribution is to provide healthcare providers with a new dataset, which is scalable for medical bots based on Transformers in Arabic. We solve one of the biggest challenges by building an essential structure for scalable medical bots in Arabic in healthcare.

Current bots approaches are grouped into generative models (Goyal et al. 2018), which generate a proper response, and retrieval models (Wu et al. 2016), which select a proper answer from a corpus. Since retrieval methods are limited to a pre-defined dataset, generative methods have become recently popular. However, traditional generation methods cannot easily generate long, diverse, informative responses (Li et al. 2015). This is called the “safe response” problem (Li et al. 2015).

Also, large-scale pre-training (Han et al. 2021) has become the mainstream approach for building open-domain dialogue systems in English (Radford et al. 2019) and Arabic (Antoun et al. 2020).

Most previous approaches were based on rule-based models (Kumar et al. 2018), or used semantic matching between question and answer by exploring various features (Shah and Pomerantz 2010). However, these approaches need high-quality data and various external resources, which are probably difficult to obtain. To take advantage of large amounts of raw data, deep learning-based approaches (Hristidis 2018) have been proposed to learn the distributed representation of question-answer pairs.

However, according to the lack of a large enough Arabic dataset which suits the deep learning approaches in the domain of Healthcare Q &A systems and bots (Hijjawi and Elsheikh 2015).

This paper introduces the largest Arabic Healthcare Q &A dataset (MAQA) collected from various websites. The dataset consists of more than 430k questions distributed into 20 medical specializations that we contribute to the research community on Arabic computational linguistics. We foretell that this large dataset would make a useful source for various NLP tasks on Modern Standard Arabic (MSA). We present the data in SQLite database. MAQA made publicly available in (Abdelhay and Mohammed 2022).

MAQA is the largest, to our knowledge, available and representative Q &A Arabic dataset suitable for Healthcare Q &A and bots, as well as other NLP tasks (Elnagar and Einea 2016). It also offers up to twenty main distinct categories, which are appropriately selected to eliminate ambiguity, making it robust for accurate text categorization. Also, it offers more than 430k discrete questions, which are appropriately selected to eliminate ambiguity and make it robust for accurate healthcare Q &A systems and bots tasks (Almansor and Hussain 2020).

Also, in contrast with the few small available datasets (Elnagar and Einea 2016), MAQA’s size makes it a suitable corpus for implementing both classical and deep learning models (Antoun et al. 2020). Also, the paper uses the constructed corpus to build three deep learning approaches using long short-term memory (LSTM) (Graves et al. 2006), Bi-LSTM (Clark et al. 2018), and Transforms (Wael et al. 2021). With the help of a pre-trained continuous bag of words (CBOW) wording embedding technique (Mohammad et al. 2017), a comparative evaluation of three models will also be presented.

The rest of the paper is organized as follows: Sect. 2 summarizes the related work done in this field, particularly those works that applied deep learning techniques. Section 3 introduces the proposed corpus. Section 4 describes proposed deep learning models. Section 5 shows the experimental results and the evaluation of the proposed deep learning models on the proposed corpus. Finally, Sect. 7 concludes the paper. Moreover, Appendix 1 shows information about bot deep learning models and evaluation methods.

## Related work

There are many deep learning research efforts focused on English bots and text generations. Many papers have used LSTM. For example, (Rarhi et al. 2017) created a bot using AIML (Artificial Intelligence Mark-up Language) to answer questions in the context of medical issues and symptoms queries to redirect the user to the correct doctor, using term detection ratio to evaluate their work which achieved a score of 56.6%.

Moreover, the authors of (Wu et al. 2018) proposed a network architecture called TACNTN to select the answer from the knowledge base according to the question topic. They used a pre-trained LDA model to obtain the topic weights. They used Ubuntu Corpus for English plus the posts crawled from Sina Weibo for Chaines, and their work approved significant accuracy in message-response matching.

Also, the work in deep learning-based bot (Csaky 2019) applied LSTM on Cornell Movie Dialog Corpus and OpenSubtitles Corpus. The corpus is a multi-turn dialogue, and the context is related to the movie genre; they achieved a BLeU score of 47% using a dataset containing 220k pairs. Similarly, the others of (Athota et al. 2020) created a retrieval healthcare bot, and they used cosine similarity to match and evaluate their work, achieving a cosine similarity of 85.6%.

Unlike, in (Bao et al. 2020), the authors proposed a framework to create healthcare question-answering systems using a knowledge graph and Bi-LSTM network. They used the matching score to evaluate their work which achieved a score of 81.02%.

However, only some previous approaches worked with Arabic datasets. On the other hand, the authors of (Boulesnane et al. 2022) created a medical assistant bot using a dataset containing 2150 pairs in Arabic-Algerian accents. They used LSTM Architecture and the Fraction of relevant as a primary metric in their work which achieved 90%. Similarly, the paper (Naous et al. 2021) proposed an LSTM model for the Arabic Empathetic bot, which was applied in a dataset of  38K samples that achieved a BLeU score of 50%.

However, the previous researchers were outside the context of medical and healthcare advice systems. The work of (Habib et al. 2021) is a collaboration between a popular medical website to provide medical advice and some leading universities which relied on actual data for the highest medical specialties for which counseling has been requested. They used a combination of LSTM and CONV1D to train at two versions on 3-gram and 4-gram datasets. They have achieved a matching score of 40.6%.

Also, the authors of (Wael et al. 2021) built a question-answering system for medical questions using Bi-LSTM+Attention and BERT models. They achieved an accuracy rate of 80.43% at their proposed English corpus.

Beside, the work of (Kora and Mohammed 2023) is providing an annotated dataset which contains 50K of Arabic tweets, and new ensemble approach to enhance the sentiment analysis in Arabic. Table 1 shows the summary or related work. Also, Table 2 shows a comparison between our dataset (MAQA) and the other datasets, which indicates that the MAQA dataset is the largest Arabic dataset in the healthcare domain.

Despite that, all previous work has used small datasets, as shown in Table 2. Thus, the MAQA dataset provides the largest Arabic dataset in the healthcare Q &A context.

**Table 1:** Summary of related works

References	Year	Language	Task	Evaluation metrics
Rarhi et al. (2017)	2017	English	Medical QA	Term detection ratio 56.6%
Wu et al. (2018)	2018	English	Single turn bot	BLeU score 61.2%
Csaky (2019)	2019	English	multi-turn bot	BLeU score 47%
Athota et al. (2020)	2020	English	Retrieval bot	Cosine similarity 85.6%
Bao et al. (2020)	2020	English	Single turn bot	Matching score 81.2%
Naous et al. (2021)	2021	Arabic	Empathetic bot	BLeU score 55.8%
Habib et al. (2021)	2021	Arabic	Medical Recommendations	Matching score 40.6%
Wael et al. (2021)	2021	Arabic	Text classification	Accuracy 95%
Boulesnane et al. (2022)	2022	Arabic	Single turn bot	Fraction of relevant 90%
Kora and Mohammed (2023)	2023	Arabic	Sentiment analysis	Accuracy 80.3%

**Table 2:** Comparison of datasets

Dataset	Task	Size	Metrics	Model	Metric value
MAQA (Abdelhay and Mohammed 2022)	MedicalBot	430,000	BLeU	Transformer	0.56
ASMCHA (Alayba et al. 2017)	Sentiment analysis	126,959	Accuracy	CNN	0.9
Arabic empathetic dialogues (Naous et al. 2021)	Empathetic bot	36,628	BLeU	BERT	0.558
Private dataset (Habib et al. 2021)	Medical recommendations	36,628	Matching	Bi-LSTM	0.406
Private dataset (Wael et al. 2021)	Text classification	N/A	Accuracy	Bi-LSTM	0.95
DZchatbot (Boulesnane et al. 2022)	Chatbot	81,659	Accuracy	GRU	0.95
Corpus on Arabic Egyptian tweets (Kora and Mohammed 2019)	Sentiment analysis	50,000	Accuracy	Meta-ensemble	0.853

## Proposed Arabic corpus

There are few Arabic datasets for Q &A tasks, and correctly categorize them into several domains compared to other languages, such as English. In this section, we propose a corpus of healthcare Arabic Q &A. Before that, let us discuss the characteristics of the Arabic language.

### Characteristics of Arabic

We can elaborate most common characteristics of Arabic as follows:

- Arabic is one of the most widely used languages in the world.
- Arabic is one of the UN’s six official languages according to (Vilares et al. 2017).
- Arabic is a Semitic language, and its letters in order include 28 letters from right to left. The letter sets have diverse composed structures as indicated by their position in words, whether toward the start, center, or end. For example, the letter (**ي**), pronounced as yaa, is written as (**يـ**) if it comes at the beginning of a word, and it is written as (**ـيـ**) if it comes in between the letters, whereas it is written as (**ـي**) at the end of the word (AlOtaibi and Khan 2017).
- The Arabic language has three primary structures: old style, present-day standard what is more, and causal structure. The traditional structure is the language of the composed Quran (IslamHoly Book). The Arabic Standard Advanced Structure (MSA) is widespread in Arab nations and is the compound language used in formal correspondence, writing books, and documents. The informal structure is spoken and composed casually between most people in everyday correspondence (AlOtaibi and Khan 2017).
- Composed words in present-day standard or informal structures sometimes need to give more data about their right significance or elocution. These words are either comprehended from the specific situation or explained with diacritics to clarify their phonetics and significance. For model, the word (**شِعْرٌ**) translated as poetry and the word (**شَعْرٌ**) translated ass hair; both have same written from (**شعر**). This composed structure alone without setting is confounding in articulation or importance. Normally, present-day standard Arabic and conversational Arabic do not utilize diacritics to explain the words, yet words are perceived from the context of the written text (AlOtaibi and Khan 2017).

### Corpus

Bot and Q &A are among the hottest topics in natural language processing (NLP) tasks. The bot is getting more important nowadays, although it has less attention in Arabic than in English research due to the need for large enough datasets. Therefore, we introduce the largest Arabic Healthcare Q &A dataset as we know. (MAQA) was collected from various websites which are listed in Table 3.

**Table 3:** Scraped websites

Website	Percent
altibbi.com	70
tbeeb.net	20
cura.healthcare	10

The dataset consists of more than 430k questions distributed into 20 medical specializations that we contribute to the research community on Arabic computational linguistics. This large dataset would make a valuable source for various NLP tasks on Modern Standard Arabic (MSA). We present the data in SQLite database, MAQA is made publicly free available in (Abdelhay and Mohammed 2022). MAQA corpus is a considerable collection of Arabic Q &A in healthcare written in MSA. MAQA can be used in several NLP tasks, such as bot (Hijjawi and Elsheikh 2015), question answering, text classification, and producing word embedding models (Mohammad et al. 2017). The dataset has 430k questions organized into twenty categories, which map to medical specializations such as Pediatric, Blood diseases, Esoteric diseases, Plastic surgery, General Surgery, and Dentist (altibbi 2020).

The first stage of building the corpus is to collect an appropriate bag of questions. In this initial stage, a collection of 649,128 noisy raw questions were gathered. This collection contains questions written in MSA and some of them are written in different dialects. The second stage of building the corpus was filtering and prepossessing the questions. In this stage, a manual selection of questions is performed to identify those questions written in Egyptian dialect and MSA form. Each selected question is adapted to the medical specializations as its label. Along with the selection steps, a manual cleaning and processing phase is performed. The cleaning steps include removing repeated questions, links and hashtags, emojis, and non-Arabic letters from the questions. Also, the prepossessing phase is manually performed to do the following:

1. Remove duplicated questions that were retrieved multiple times.
2. Remove questions that contain any advertisements or inappropriate links.
3. Remove any special characters and none Arabic letters, particularly the letters that are similar to the Arabic letter style.
4. Remove any diacritics of word diacritics such as (**العَدَّدُ** means count) and (**العُدَدّ** means tools).
5. Remove elongation (unnecessary repeated characters) from a word such as (**جــــــدا** means very) becomes **جدا**.
6. Standardizing letters forms of the words, for example, replace the different forms of the latter Alif written as (**أ**, **آ**, **إ**) by bare Alif (**ا**), replace the letter (**ى**) at the end of a word by the letter (**ي**), replace the letter (**ة**) at the end of a word by the letter (**ه**), replacing the different forms of the letters (**ؤ**,**و**) by the letter (**و**), and finally replace the form of the letter (**ي**,**ئ**) by (**ي**),and replacing the different forms of (**لإ**,**لآ**,**لأ**) into (**لا**)
7. Normalize words that are combined together by adding spaces between words.
8. Finally, manually correcting words that have missing letters, contain wrongly replaced letters, or are wrongly written.

**Fig. 1:**
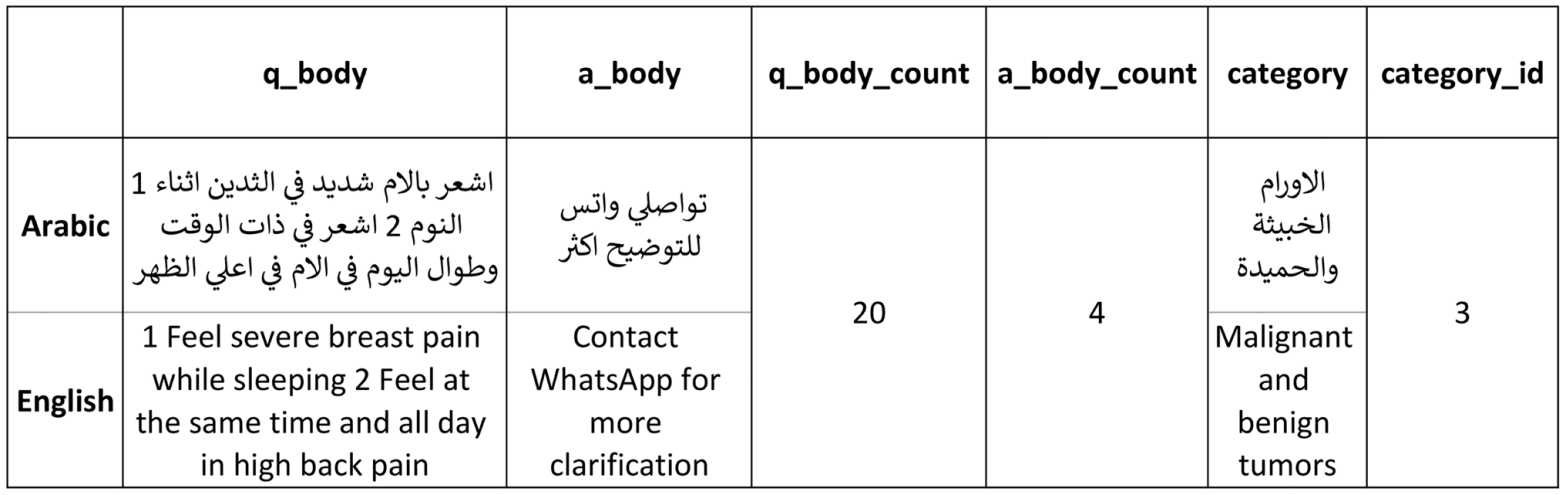
An example of a Question

After applying the selection and preprocessing step, we have 434,543 questions categorized and formatted as shown in Fig. 1. The statistics of the corpus are shown in Table 4.

**Table 4:** MAQA corpus statistics

Total number of questions	434,543
Number of words	33,847
Max question token	100
Max answer token	100
Number of tokens	10,128,624
Average tokens per question	23
Average tokens per answer	19

In general, MAQA adopted the annotation of each question as appeared in its website source (altibbi 2020). The distribution of questions per category is summarized in Table 5.

**Table 5:** Distribution of questions per category

Label	Count
Gynecology diseases	103,683
Urogenital diseases	33,847
Musculoskeletal and joint diseases	33,050
Dermatology diseases	29,262
General medicine	26,870
Esoteric diseases	23,722
Gastrointestinal diseases	22,373
Sexually transmitted diseases	21,773
Dentistry	20,207
Pediatric	18,636
Psychiatric and neurological diseases	18,295
Cardiovascular disease	15,368
General surgery	15,185
Ophthalmology	14,439
Ear nose and throat—ENT	13,933
Malignant and benign tumors	11,210
Endocrine diseases	5186
Respiratory system diseases	4567
Plastic surgery	1596
Blood diseases	1341

The data has stored in SQLite database in a table called ds5b which contains the following columns in Table 6:

**Table 6:** Dataset’s columns and description

Label	Description
q_body	Contains the question content
a_body	Contains the answer content
q_body_count	Contains the question content word count
a_body_count	Contains the answer content word count
category	Contains category name
category_id	Contains category number from table categories

All questions are unique. The data is kept in raw format, cleaned but not stemmed, or any other preprocessing has applied after scraping. The questions and answers contain some English symbols and digits; and almost no Arabic diacritics or punctuation. Figure 1 shows an example of a question from the “Malignant and benign tumors” category.

The distributions of all questions per category (aka label) regarding count and percentages are depicted in Table 7. The detailed number of questions per category is shown in Table 5. We came up with the MAQA abbreviation as first letters of Medical Arabic Questions and Answers. The questions and their answers were collected using Python scripts written specifically for scraping popular medical and healthcare question-answering portals (altibbi 2020). Those scripts load the list of the portal’s questions, enter each question’s page, and get its text, answer, and specialization (aka category). The data collection procedures are described below:

The main website has a portal for users to ask their questions and get them answered by the doctors. After scraping the questions and their answers, we grouped them into categories that adapted the same specialization, or clinic name of the questioning portal (altibbi 2020).

After examining the content of categories, some categories have been manually merged with some other categories, as “sexual health” and “infertility” have merged into “sexual diseases,” and “Hypertension” has merged into “Esoteric diseases” for the full list of merged categories show Table 7.

**Table 7:** Manually merged categories into other categories

Original category	Merged into category
Sexual health	Sexual diseases
Pregnancy and childbirth	Gynecological diseases
Preventive medicine	General medicine
Alternative medicine	General medicine
Gynecological surgery	Gynecological diseases
Infertility	Sexual diseases
Urology	Urology and genitourinary diseases
Hypertension	Esoteric diseases
Cardiovascular surgery	Cardiovascular disease
Dental and jaw surgery	Dentistry
Orthopedics	Musculoskeletal and joint diseases
Infectious diseases	General medicine$^{2}$

We collected a set of 430k questions since January 1, 2019 until January 1, 2020. We have scraped three popular websites which are declared in Table 3. Moreover, we applied clean and merged some categories and the resulting distribution of the twenty categories (Table 5).

## Proposed approaches

This section proposes three deep learning models on the corpus to generate answers to input questions automatically. Specifically, the paper propounds LSTM, Bi-LSTM, and Transformers models. They are among the popular models used in the literature for deep learning NLP tasks, particularly in text generation and bots (Reddy Karri and Santhosh Kumar 2020). Long short-term memory (LSTM) is an artificial neural network used in AI and deep learning. Unlike standard feedforward neural networks, LSTM has feedback connections. Such a recurrent neural network (RNN) can process individual data points (such as an article) as well as entire data sequences (such as speech or document) (Sak et al. 2014). LSTM deals with both long-term memory (LTM) and short-term memory (STM) and uses the concept of gates to make computation simple and efficient Fig. 8.

- Forget Gate: LTM moves to forget gate and forgets the useless information.
- Learn Gate: Events (current input) and STM are combined with applying the desired information recently learned from STM to the current input.
- Remember Gate: The remembered LTM information and STMs and events are combined into a remember gate that acts as an updated LTM.
- Use Gate: This gate uses the LTM, STM, and Event to predict the output of the current Event, which acts as an updated STM.

Although long short-term memory (LSTM) network has become a successful architecture that handles the sequence of text (Lyu and Liu 2021), yet LSTM-based encoder/decoder models do not work well for long sentences. This is because such sentences have a single latent vector as output, and the final LSTM unit may not capture the entire essence of the sentence. Since all words in a long set are captured in one vector, naive LSTM-based encoder/decoder models should care better if the output words depend on specific input words. This is where the attention-based model came into existence (Vaswani et al. 2017).

The Transformer model is an attention-based model, where the decoder has access to all past states of the encoder instead of relying solely on the context vector; that is how attention works. At each decoding step, the decoder can see the specific state of the encoder.

**Fig. 2:**
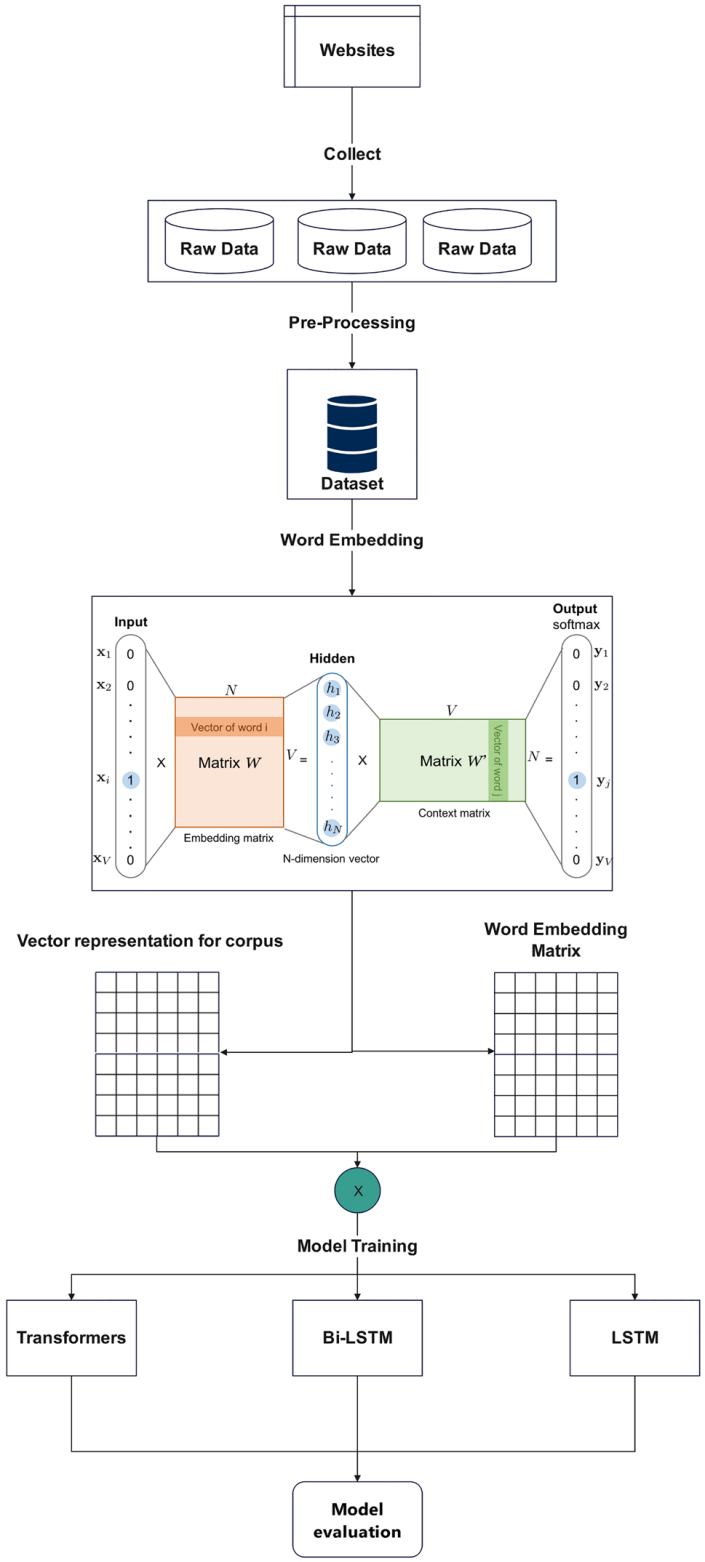
General pipeline architecture of the proposed models

The general architecture of our models is depicted in Fig. 2. Each model begins with an input layer followed by the layers of its network architecture. The input layer is a generic word vector representation by word2vec embedding model (Mikolov et al. 2013). CBOW predicts target words from context words and skip-gram predicts contextual words from target words. among them The proposed deep learning model uses trained word vectors In Word2vec CBOW, especially pre-trained CBOW model called Aravec (Mohammad et al. 2017). This model was trained on 132,750,000 Arabic documents with 3,300,000,000 words. A word embedding matrix is then generated based on the pre-trained CBOW model. The word embedding sentences of the corpus generated by the word embedding matrix are then fed to each network as input features. The layers of each network are described in Appendix 1. Also, the pseudocode of the training is as following:

- Initialize the LSTM model with randomly initialized parameters
- Split the dataset into training and validation sets
- Define the loss function, such as cross-entropy loss, and the optimizer, such as Adam or SGD
- Set the number of training epochs and the batch size
- For each epoch:
  1. Shuffle the training set
  2. Split the training set into batches of size batch_size
  3. For each batch:
    - Encode the input text using an embedding layer
    - Pass the embeddings through the LSTM layers
    - Compute the output logits using a linear layer on top of the LSTM layers
    - Compute the loss between the predicted logits and the true labels
    - Backpropagate the loss and update the model parameters using the optimizer
  4. Compute the validation loss on the validation set and save the model if it achieves the lowest validation loss so far
- Test the model on a held-out test set and evaluate its performance using appropriate metrics, such as cosine similarity and BLeU score.

### Working environment

Regarding the hardware settings of the experiments, the development platform was Google Colaboratory (Colab). Regarding the Colab, the processor was Intel(R) Xeon(R) CPU @ 2.00GHz, and the memory was 27 GB. Regarding the cloud server, the Python version 3.7.3 was used on Ubuntu-1804-bionic-64 cloud server, the memory was 64 GB, and the processor was an Intel(R) Core(TM) i7-7700 with a speed of 3.6 GHz. Meanwhile, the used GPU was GeForce GTX 1080 (8 GB). Furthermore, the utilized deep learning framework was PyTorch (Imambi et al. 2021). Besides, the utilized embedding models are the PyTorch embedding, and the Aravec-WWW-CBOW at dimension 300 (Mohammad et al. 2017).

### Performance parameter for evaluation

There are three major methods to measure bots’ accuracy and generative models. Cosine similarity (Zhou et al. 2022), BLeU score (Papineni et al. 2002), and Perplexity (Meister and Cotterell 2021) are three important metrics used to measure the effectiveness of a bot. We chose to compare cosine similarity as in equations from A4 to A7. BLeU score as in equation from A8 to A13 of our proposed models, hence the cosine similarity has a problem with similarity of high frequency words (Zhou et al. 2022).

## Experimental results

This section will present the experimental results of LSTM, Bi-LSTM, and Transformers model on our proposed corpus. We train and test each model using splits of (70%, 20%, 10%) for the train, validation, and test dataset, respectively. We merged some categories (sub-specializations) into their main categories. We removed the pairs containing mixed languages code-mixing (CM), a common problem in most social media platforms (Dowlagar and Mamidi 2021). After all, preprocessing, there were 450,000 pairs left; from them, we sampled all pairs with a maximum length of 30 words for both question and answer, which was 254,588 distinct question-answer pairs. Then, we split to train, test, and validate the dataset, and we considered keeping the representation rate for each category as same as the whole dataset as shown in Table 8. We used cosine similarity and BLeU score to evaluate our models.

**Table 8:** Distribution of questions per category per dataset

Category	Train (%)	Validation (%)	Test (%)
Gynecology diseases	7.28	7.28	7.28
Urogenital diseases	4.55	4.55	4.55
Musculoskeletal and joint diseases	2.64	2.63	2.64
Dermatology diseases	7.35	7.35	7.35
General medicine	4.58	4.58	4.58
Esoteric diseases	1.00	1.00	1.01
Gastrointestinal diseases	3.36	3.36	3.36
sexually transmitted diseases	0.30	0.30	0.31
Dentistry	6.91	6.91	6.91
Pediatric	4.20	4.20	4.20
Psychiatric and neurological diseases	1.48	1.48	1.49
Cardiovascular disease	2.69	2.69	2.69
General surgery	7.17	7.17	7.17
Ophthalmology	5.50	5.50	5.50
Ear nose and throat—ENT	26.76	26.77	26.74
Malignant and benign tumors	2.77	2.77	2.77
Endocrine diseases	3.27	3.27	3.28
Respiratory system diseases	0.48	0.48	0.49
Plastic surgery	3.37	3.37	3.37
Blood diseases	4.32	4.32	4.32

We have fined-tuned several hyper-parameters for each model. Table 9 shows the best values of the hyper-parameters used in the run of the three models. Moreover, Table 10 shows the different hyper-parameters we have used in earlier experiments.

**Table 9:** Hyper-parameters values for LSTM, Bi-LSTM, and Transformers models

Models	Learning rate ($\alpha$)	Batch size	Dropout	Epochs
LSTM	0.001	128	0.2	100
Bi-LSTM	0.001	128	0.3	100
Transformers	0.001	128	0.2	100

**Table 10:** Early experiment hyper-parameters values for LSTM, Bi-LSTM, and Transformers models

Hyper-parameter	Run 1	Run 2	Run 3
Learning rate ($\alpha$)	1e–5	5–e4	0.001
Batch size	28	64	128
Dropout	0.4	0.3	0.2
Epochs	200	150	100
Maximum sequence length	100	50	30
Units	1024	512	512
LSTM layer	2	6	10
Number of Heads	4	8	10
Vocab size	75,000	50,000	32,768
AVG loss	N/A	3.7	0.9
AVG cosine similarity	N/A	42%	80%
AVG BLeU score	N/A	22%	58%

In addition to the hyper-parameters, Table 11 shows the latest configuration of the LSTM model. In this configuration, the sequence length is 30 according to computation limits, representing about 60% of the dataset. In other words, the number of words representing the largest pair selected is 30. In addition, vocab size is the number of words that enter the network each time, and it is selected to be 32,768 words.

**Table 11:** Configuration of LSTM parameters

Sequence length	30
Units	512
LSTM layer	10
Vocab size	32,768

Similarly, Table 12 shows the latest configuration of the Bi-LSTM model. In this configuration, 10 LSTM layers with 1024 LSTM cells have been applied.

**Table 12:** Configuration of Bi-LSTM parameters

Sequence length	30
Units	1024
LSTM layer	10
Vocab size	32,768

Table 13 shows the latest configuration of the Transformers model. This model uses the same configuration with multi-head attention with 10 Heads (Vaswani et al. 2017).

**Table 13:** Configuration of Transformers parameters

Sequence length	30
Units	1024
Number of heads	10
Vocab size	32,768

Table 14 summarizes the result of all models. The results show that the Transformers’ average cosine similarity is higher than the other models at a rate between $9.00\%$ and $10.00\%$. Also, the training time has increased at a rate of $25\%$. Also, we noted that the BLeU score is not a proper metric for Arabic.

**Table 14:** Results of models

Model	Average cosine similarity	Average BLeU score
LSTM	0.56	0.31
Bi-LSTM	0.72	0.39
Transformers	0.80	0.58

### Discussion

Figure 6 shows the comparison of training scores per 100 epochs for models. Generally, in the training process within deep learning using stochastic gradient descent, the scores are measured per batch and tend to oscillate up and down. We do not normally get a monotonic increase in scores within each batch. The normal way to handle the scores is to average overall batches per epoch.

**Fig. 3:**
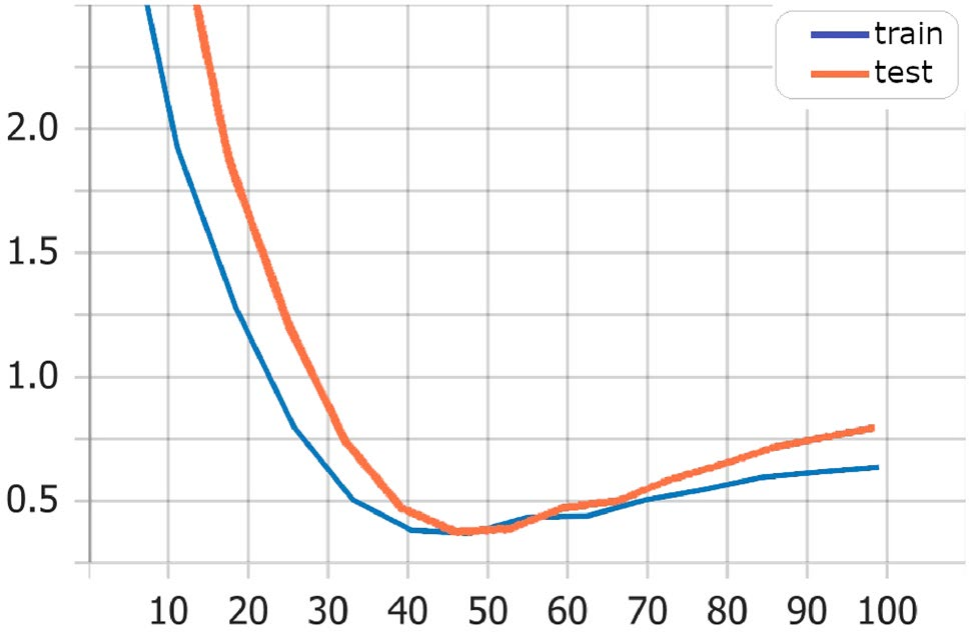
Transformers model training versus test loss

**Fig. 4:**
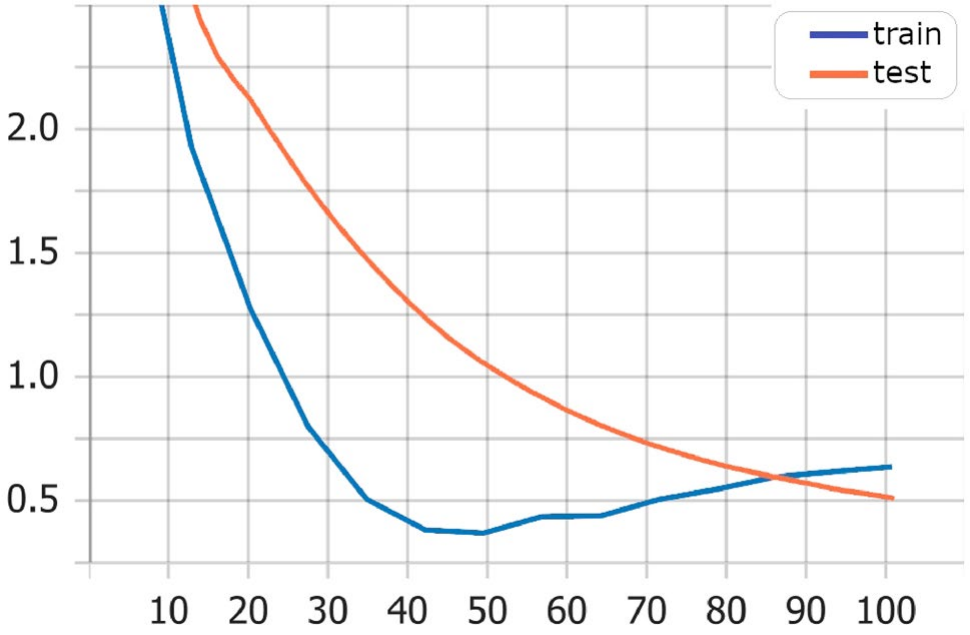
LSTM model training versus test loss

**Fig. 5:**
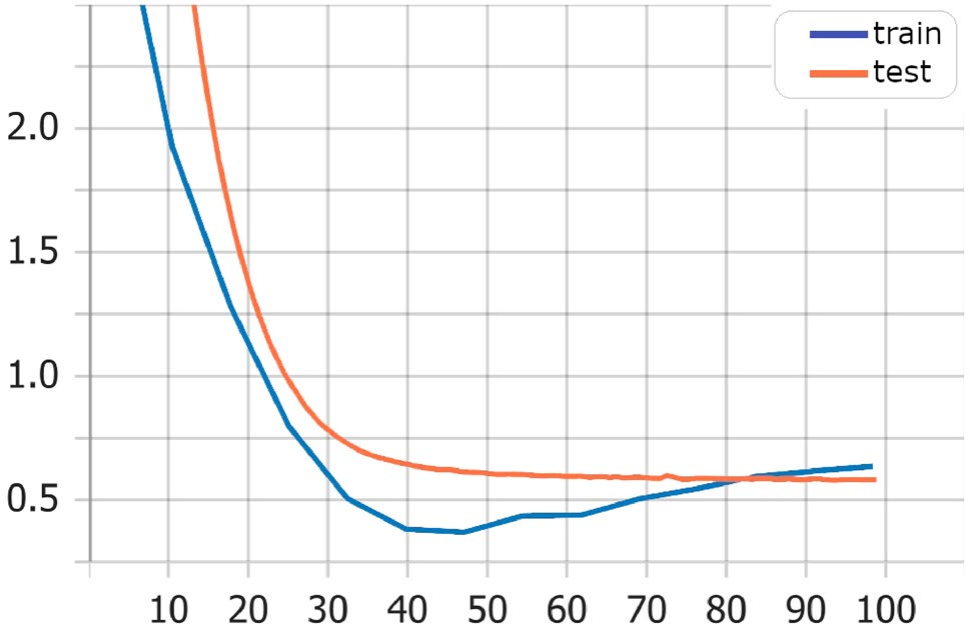
Bi-LSTM model training versus test loss

Also, training and test loss show that the Transformers model has best values Fig. 3 in comparison with LSTM model Fig. 4, and Bi-LSTM model Fig. 5.

**Fig. 6:**
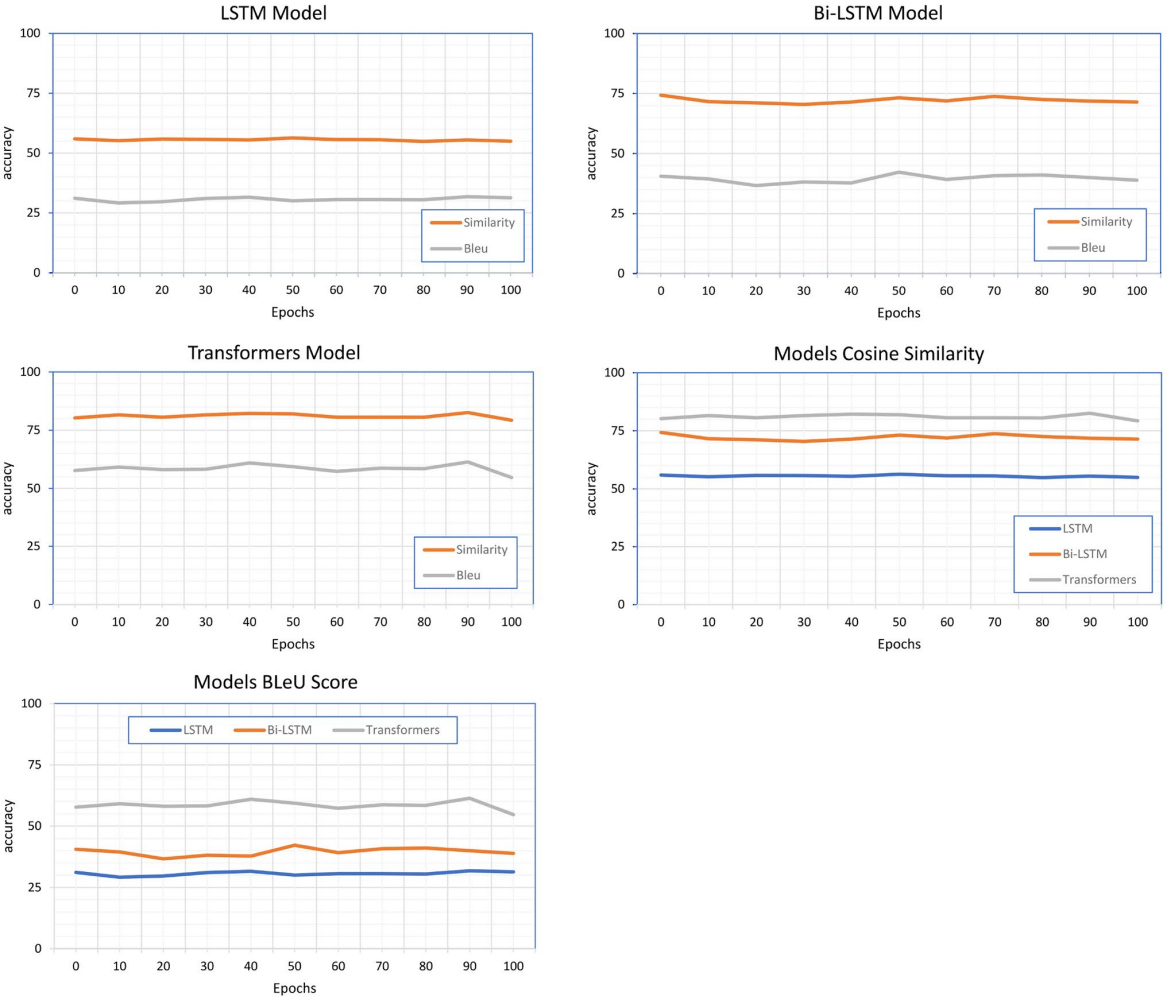
Average training similarity and BLeU per 100 epochs for various models

**Fig. 7:**
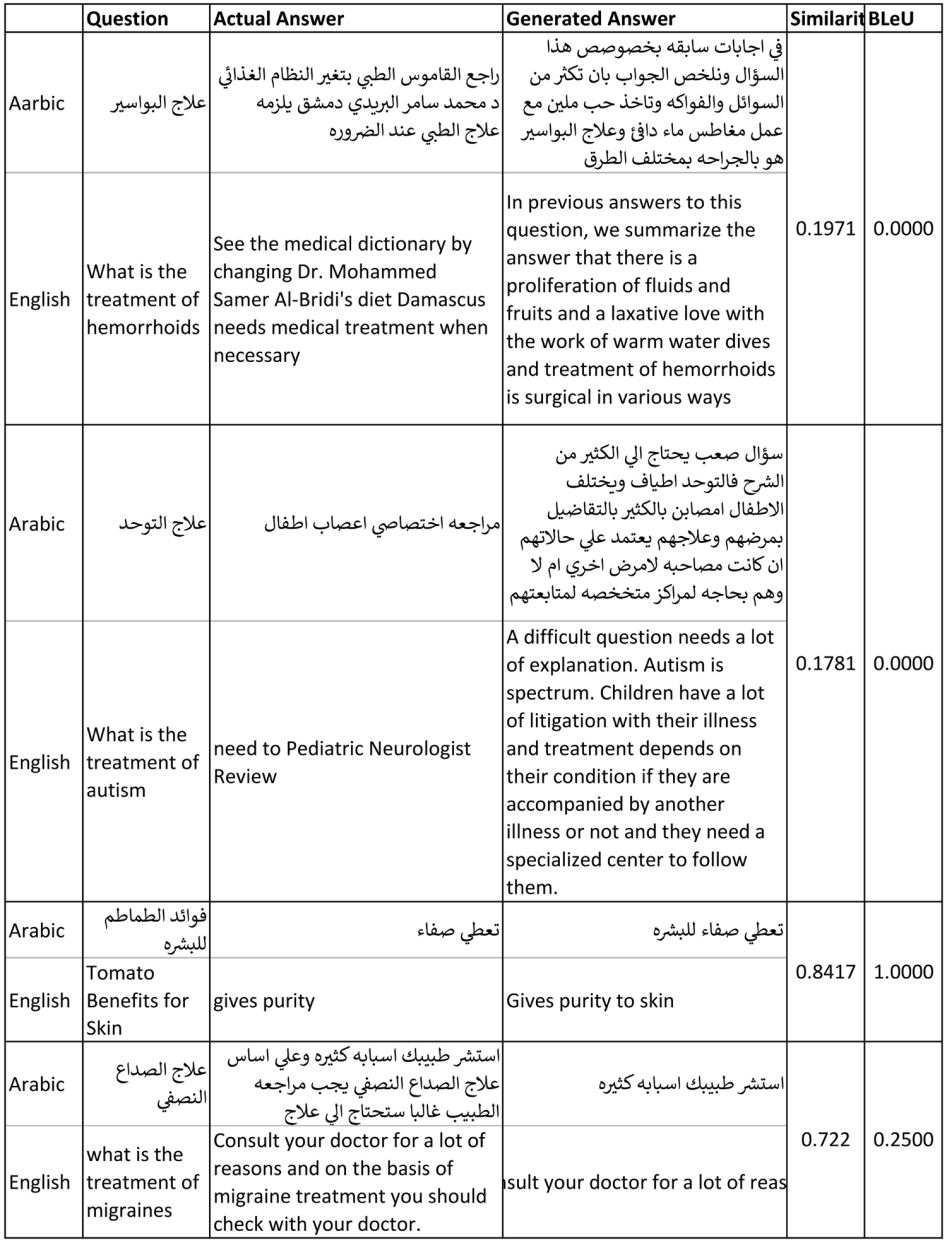
Sample of testing data with generated output and their scores

**Fig. 8:**
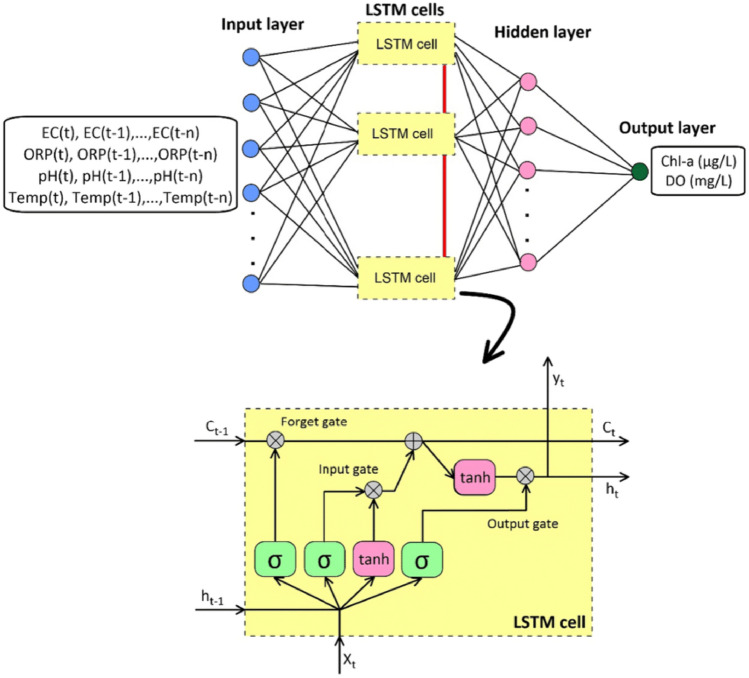
LSTM model architecture and structure of LSTM cell

The results of training average performance using cosine similarity and BLeU score show that the BLeU score is more sensitive for using different synonymous in output sentences than the actual one (Fig. 6). The cosine similarity gives us a more convenient metric, yet it still shows a sensitivity against different synonymous and insensitivity of the context. Figure 7 shows examples of testing data with actual and generated output, showing that the cosine similarity is less than 20%. However, according to human judgment, the generated output is relevant and strongly related to the answer.

So, to gain more useful insights into the similarities, we ran some statistics summarized in Table 15. From statistics in Table 15, we get the Transformers model outperforms the other models and performs a similarity rate at 84.4% for 75% of samples. Also, Table 16 shows the same statistics applied to the BLeU score, which shows that the 75% of samples achieved a score of 94%.

**Table 15:** Statistics of models output similarities

	LSTM	Bi-LSTM	Transformers
mean	0.565542	0.573685	0.689978
std	0.246773	0.249254	0.297342
min	0.000000	0.000000	0.000000
25%	0.256600	0.203200	0.300000
50%	0.505300	0.560400	0.637000
75%	0.597900	0.616500	0.843775
max	1.000000	1.000000	1.000000

**Table 16:** Statistics of models output BLeU score

	LSTM	Bi-LSTM	Transformers
mean	0.311251	0.391983	0.580030
std	0.167223	0.248062	0.322365
min	0.100000	0.100000	0.100000
25%	0.170000	0.200000	0.250000
50%	0.250000	0.290000	0.570000
75%	0.500000	0.560000	0.940000
max	1.000000	1.000000	1.000000

## Limitations

There are several limitations to the current study that need to be acknowledged. Firstly, the proposed dataset was collected from a specific social media platform and may not be representative of the broader Arabic language used in other contexts. Secondly, the study only experimented with three deep learning techniques, namely LSTM, Bi-LSTM, and Transformers, and other deep learning architectures were not explored. Thirdly, while the performance of the deep learning models on the proposed corpus was promising, the evaluation metrics used, namely cosine similarity and BLeU score, are sensitive to outlier values and different synonymous, which may limit the results generalization. Also, tthe proposed corpus, although large, may still not be sufficient for some conversational agents (bots) applications, particularly those that require more specific domain knowledge or rare categories.

Finally, the experiments were conducted using a single GPU, which limited the batch size and training time for the deep learning models. Additionally, the software used for data prepossessing and model training had its own limitations and could have potentially impacted the results. Future studies could benefit from using more powerful hardware and software to potentially improve the performance of the models.

## Conclusion

Recently, deep learning methods have shown a significant impact and powerful techniques in various applications like machine translation, speech recognition, computer vision, and NLP. Lately, applying deep learning techniques to bots has become increasingly popular, especially after the hype of ChatGPT, outperforming standard machine learning algorithms. Thus, many researchers applied deep learning techniques to CA (bot) tasks in several spoken languages. Arabic is one of the most widely used languages in the world and is used extensively on social media with different forms and dialects. However, one limitation to applying deep learning techniques to Arabic bots is the availability of suitable large corpora. Thus, this paper introduced a labeled corpus of 430K of Arabic Q &A into 20 different categories.

Also, the study applied three deep learning techniques to the proposed dataset. Mainly, the study experimented with the performance of the dataset on LSTM, Bi-LSTM, and Transformers. With the help of the word embedding technique as the input layer to the three models, the Transformers achieved an average cosine similarity score of 80% and an average BLeU score of 58%, outperforming LSTM with an average cosine similarity score of 56% and an average BLeU score of 31%, and Bi-LSTM with an average cosine similarity score of 72% and an average BLeU score of 39%. Applying a pre-trained word embedding showed a further improvement in both cosine similarity and BLeU score.

Since the performance of deep learning models was promising according to the proposed corpus, it is worth investigating other deep learning architectures. Also, hence the average cosine similarity and average BLeU show sensitivity to outlier values and different synonymous, we think creating a new measure focusing on the Arabic may be worth investigating to address these issues. The proposed corpus, along with the deep learning techniques applied, could contribute to the development of Arabic bots and potentially other NLP applications.

## Figures and Tables

**Fig. 9:**
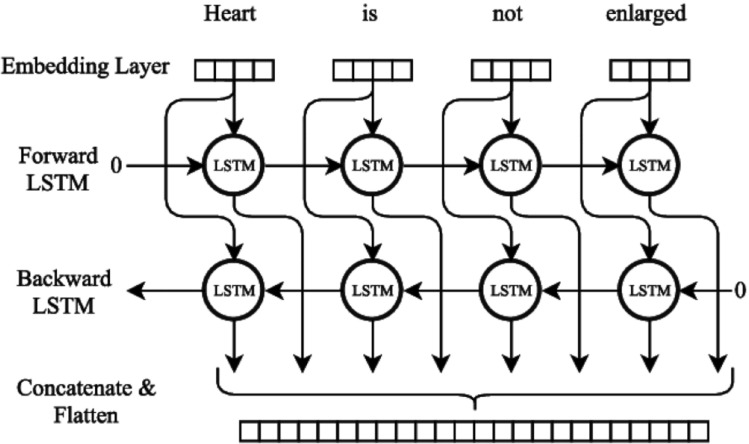
Bi-Directional LSTM model

**Fig. 10:**
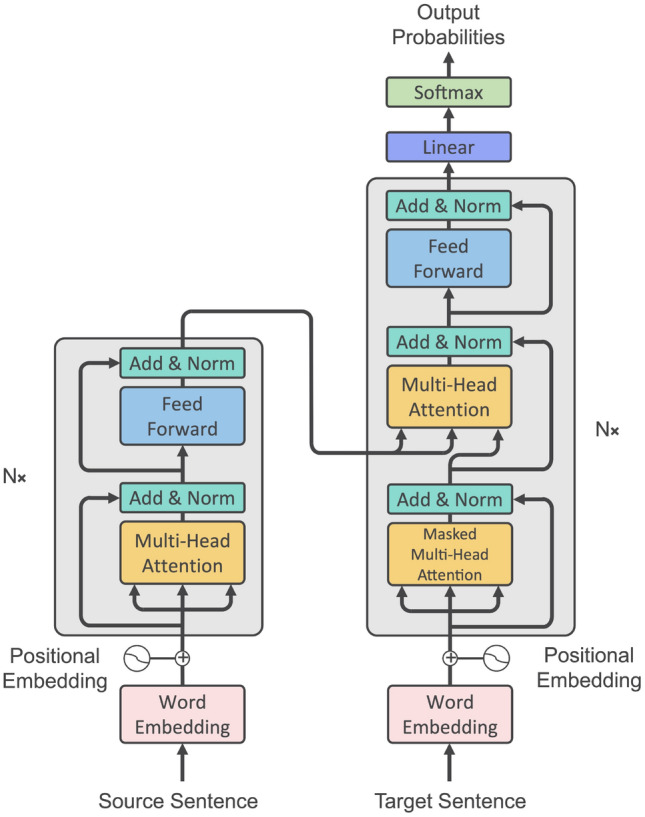
Transformers layers

## Appendix A: Preliminary

Bots are computer programs designed to simulate conversations with human users (Tascini 2019). They have used in various applications, including customer service, marketing, and entertainment. Also, task-less bots that respond intelligently to humans on diverse topics are attracting much interest from academics and industry (Wu et al. 2018b). There are several types of bots, each with its unique capabilities and features.

1. Rule-Based bots: Rule-based bots use pre-defined rules and algorithms to respond to user queries (Thorat and Jadhav 2020). They are programmed with keywords and phrases that they recognize and respond to accordingly. These bots can be used for simple tasks such as providing basic information or helping customers find what they need on a website.
2. AI bots: AI bots use artificial intelligence (AI) technology to understand user queries and provide more accurate responses (Tascini 2019). These bots can learn from their user interactions and adapt their responses accordingly. AI bots can be used for more complex tasks, such as providing customer support, making product recommendations, or helping customers complete transactions online.
3. Natural Language Processing (NLP) bots: NLP bots use natural language processing (NLP) technology to understand user queries and provide more accurate responses than rule-based bots (Paliwal et al. 2020). NLP bots can understand the context of conversations, allowing them to provide more personalized responses that better meet the user’s needs.
4. Voice bot: Voice bot uses voice recognition technology to interact with users through voice commands or natural language processing (NLP) (Wijaya and Wicaksana 2019). These bots can be used for tasks such as providing customer support or making product recommendations based on voice commands given by the user.
5. Social Media bot: Social media bot is a type of bot that interacts with users through social media platforms, such as Facebook Messenger, Twitter, and Instagram, allowing them to provide customer support or make product recommendations based on conversations held on these platforms (Sarosa et al. 2020).

All previous types are grouped into two groups, generative models (Goyal et al. 2018), which generate a proper response, and retrieval models (Wu et al. 2016), which select a proper answer from a corpus. Since retrieval methods are limited in understanding the context and generating appropriate responses, generative methods became recently popular. Moreover, with the rise of deep learning methods such as LSTM, generative bots gained more attention, and we chose three models to run against our dataset, namely LSTM, Bi-LSTM, and Transformers.

### A.1 LSTM

The LSTM consists of a memory cell, input, output, and forget gates (Han and Moraga 1995). The memory cell is responsible for remembering values over time, while the other gates control the flow of the information from and out of the cell. Figure 8 shows the layers of the LSTM model and the structure of the LSTM cell. After applying the embedding word layer, the embedded words are fed to the LSTM cells. The LSTM cells will be trained on the embedded words and produce their prediction words. The prediction words are fully connected with a dense layer of type sigmoid (Han and Moraga 1995) function.

The compact shapes of the equations for the feed-forward pass of an LSTM cell with a forget gate are shown in equation A1.

A1 $$\begin{aligned} {\begin{aligned}f_{t}&=\sigma _{g}(W_{f}x_{t}+U_{f}h_{t-1}+b_{f})\\i_{t}&=\sigma _{g}(W_{i}x_{t}+U_{i}h_{t-1}+b_{i})\\o_{t}&=\sigma _{g}(W_{o}x_{t}+U_{o}h_{t-1}+b_{o})\\{{\tilde{c}}}_{t}&=\sigma _{c}(W_{c}x_{t}+U_{c}h_{t-1}+b_{c})\\c_{t}&=f_{t}\odot c_{t-1}+i_{t}\odot {{\tilde{c}}}_{t}\\h_{t}&=o_{t}\odot \sigma _{h}(c_{t})\end{aligned}} \end{aligned}$$

where the initial values are $c_{0}=0$ and $h_{0}=0$ and operator $\odot$ the Hadamard product (element-wise product) (Horn and Yang 2020). The subscript *t* indexes the time step. More, the used variables are:

- $x_{t}\in {\mathbb {R}} ^{d}$: input vector to the LSTM unit.
- $f_{t}\in {(0,1)}^{h}$: forget gate’s activation vector.
- $i_{t}\in {(0,1)}^{h}$: input/update gate’s activation vector.
- $o_{t}\in {(0,1)}^{h}$: output gate’s activation vector.
- $h_{t}\in {(-1,1)}^{h}$: hidden state vector also known as output vector of the LSTM unit.
- ${{\tilde{c}}}_{t}\in {(-1,1)}^{h}$: cell input activation vector.
- $c_{t}\in {\mathbb {R}} ^{h}$: cell state vector
- $W\in {\mathbb {R}} ^{h\times d}$, $U\in {\mathbb {R}} ^{h\times h}$, and $b\in {\mathbb {R}} ^{h}$: weight matrices and bias vector parameters which need to be learned during training where the superscripts *d* and *h* refer to the number of input features and number of hidden units, respectively.

### A.2 Bi-LSTM

The Bi-LSTM or Bi-Directional LSTM model is a sequential processing model that consists of two LSTMs (Clark et al. 2018). One accepts forward to input, and one accepts reverse input as shown in Fig. 9. Bi-LSTM effectively increases the amount of information available to the network and improves the context available to algorithms (for example, knowing which words immediately follow and precede a word in a sentence) (Clark et al. 2018).

### A.3 Transformers

Furthermore, and finally, the Transformers model is essentially an attention-based model (Vaswani et al. 2017). Attention is a means of selectively weighing different elements in the input data to influence the hidden states of downstream layers adequately (Vaswani et al. 2017). The scalar product of key and query provides attention weights, which are compressed across all attention weights using the SoftMax function, resulting in a total weight of 1. The value vectors corresponding to each element are summed according to their attention weights before being fed to subsequent layers (Vaswani et al. 2017), as shown in Fig. 10.

The formula for the softmax function is as follows (equation A2):

A2 $$\begin{aligned} \textrm{softmax}(x) = e^x_i / \mathrm{{ sum }} \, \left( e^x_j\right) \mathrm{{ for }} j=1\, \mathrm{{ to }} \, N \end{aligned}$$

where

- *x* is a vector of input values, with *N* elements.
- *e* is the base of the natural logarithm.
- *i* is the index of the current element in the vector *x*.
- *j* is the index of each element in the vector *x*, summed over all elements.
- $sum(e^x_j)$ is the sum of the exponential values of all elements in the vector *x*

The attention scores are computed based on a dot product between the input values and a set of learnable query, key, and value vectors (Vaswani et al. 2017). The formula for computing the attention scores in the Transformer model is as follows (equation A3):

A3 $$\begin{aligned} \textrm{Attention}(Q, K, V) = \textrm{softmax}(QK^T / \textrm{sqrt}(d_k))V \end{aligned}$$

where

- *Q*, *K*, and *V* are matrices of learnable query, key, and value vectors, respectively
- $^T$ denotes the transpose of a matrix
- $/ \textrm{sqrt}(d_k)$ is a normalization factor to prevent the dot product from becoming too large
- Softmax is the softmax function applied to the dot product of the query and key matrices
- The output of the self-attention mechanism is computed as a weighted sum of the value matrix *V*, with weights determined by the attention scores

### A.4 Performance evaluation

With regard to performance evaluation, we have three main methods to measure the accuracy of bots and generative models (Chauhan and Daniel 2022). Cosine similarity (Zhou et al. 2022), BLeU score (Papineni et al. 2002), and Perplexity (Meister and Cotterell 2021) are the most important metrics used to measure the effectiveness of a bot. First, cosine similarity (Zhou et al. 2022) measures the similarity between two data points in a plane. Cosine similarity is used as a metric in various machine learning algorithms, such as KNN (Jiang et al. 2022), to determine the distance between neighbors (Chauhan and Daniel 2022). In recommender systems, it used to recommend movies with the same similarity, and in the case of text data, it used to find text similarities in the document (Zhou et al. 2022). To evaluate our generated answer against the actual answer, we start by getting the embedding vector for each word in the sentence, then get the average for all words’ vectors as in equation A4, where A is the sentence vector, *Vi* is the word vector and N the words count in the sentence. Then, we calculate the vectors product in equation A5, where A and B are two nonzero vectors can be derived by using the Euclidean dot product formula. After that we calculate the cosine similarity between both average vectors as in equation A6, where *Ai* and *Bi* are the *i*th components of vectors *A* and *B*, respectively. Finally, we calculate the Cosine Distance as in equation A7. The greater Cosine Distance, the greater the model efficiency and accuracy (Hendy et al. 2023).

A4 $$\begin{aligned} A= & {} \sum _{i=1}^N\frac{Vi}{N} \end{aligned}$$

A5 $$\begin{aligned} A.B= & {} \Vert A \Vert ~\Vert B \Vert \cos \theta \end{aligned}$$

A6 $$\begin{aligned} \textrm{cosine similarity}= & {} \frac{\sum _{i=1}^nAiBi}{\sqrt{\sum _{i=1}^nAi^2}\sqrt{\sum _{i=1}^nBi^2}} \end{aligned}$$

A7 $$\begin{aligned} \textrm{Cosine Distance}= & {} 1~-~ \mathrm{Cosine~Similarity} \end{aligned}$$

Also, the BLeU score is an algorithm for evaluating the quality of text generated using deep learning algorithms (Papineni et al. 2002); accuracy is considered the correspondence between a machine’s output and that of a human. The base stone of the BLeU score is the familiar precision measure, which is calculated by counting the number of candidate translation words (unigrams) that occur in any reference translation and then divided by the total number of words in the candidate translation as shown in equation A8 (Hendy et al. 2023). However, as in our bot task, the modified n-gram can be generalized as in equation A9 to the case: one candidate sentence and one reference sentence, where ${\hat{y}}$ is candidate sentence and *y* is one reference sentence. Then, we start with the n-gram count summation as in equation A10 (Hendy et al. 2023). This count summation cannot be used to compare sentences since it is not normalized. If both the reference and the candidate sentences are long, the count could be huge, even if the candidate is of poor quality (Hendy et al. 2023). So we normalize it as in equation A12, and equation A13 shows the final definition of BLEU, where $w:=(w_{1},w_{2},\cdots )$ is the weighting vector, and ${{\hat{S}}}:=({{\hat{y}}}^{(1)},\cdots ,{{\hat{y}}}^{(M)})$ is candidate corpus, and $S=(S_{1},\cdots ,S_{M})$ is reference candidate corpus

A8 $$\begin{aligned}{} & {} p_{n}({{\hat{S}}};S):={\frac{\sum _{i=1}^{M}\sum _{s\in G_{n}({{\hat{y}}}^{(i)})}\min (C(s,{{\hat{y}}}^{(i)}),\max _{y\in S_{i}}C(s,y))}{\sum _{i=1}^{M}\sum _{s\in G_{n}({{\hat{y}}}^{(i)})}C(s,{{\hat{y}}}^{(i)})}} \end{aligned}$$

A9 $$\begin{aligned}{} & {} p_{n}({{\hat{y}}};y)={\frac{\sum _{s\in G_{n}({{\hat{y}}})}\min (C(s,{{\hat{y}}}),C(s,y))}{\sum _{s\in G_{n}({{\hat{y}}})}C(s,{{\hat{y}}})}} \end{aligned}$$

A10 $$\begin{aligned}{} & {} \sum _{s\in G_{n}({{\hat{y}}})}C(s,y)={\text {number of n-substrings in }}{{\hat{y}}}{\text { that appear in }}y \end{aligned}$$

A11 $$\begin{aligned}{} & {} {\sum _{s\in G_{n}({{\hat{y}}})}\min (C(s,{{\hat{y}}}),C(s,y))} \end{aligned}$$

A12 $$\begin{aligned}{} & {} {\frac{\sum _{s\in G_{n}({{\hat{y}}})}\min (C(s,{{\hat{y}}}),C(s,y))}{\sum _{s\in G_{n}({{\hat{y}}})}C(s,{{\hat{y}}})}} \end{aligned}$$

A13 $$\begin{aligned}{} & {} BLEU_{w}({{\hat{S}}};S):=BP({{\hat{S}}};S)\cdot \exp \left( \sum _{n=1}^{\infty }w_{n}\ln p_{n}({{\hat{S}}};S)\right) \end{aligned}$$

## Abbreviations

AI: Artificial intelligence
AIML: Artificial intelligence markup language
BERT: Bidirectional encoder representations from transformers
BI-LSTM: Bidirectional long short-term memory
BLEU: Bilingual evaluation understudy
CA: Conversational agents
CBOW: Continuous bag of words
CM: Code-mixing
CNN: Convolutional neural network
CONV1D: 1D Convolutional neural network
GPT: Generative pre-trained transformer
GRU: Gated recurrent units
KNN: K-nearest neighbors
LDA: Linear discriminant analysis
LSTM: Long short-term memory
LTM: Long-term memory
MAQA: Medical Arabic QA
MSA: Modern standard Arabic
NLP: Natural language processing
RNN: Recurrent neural network
STM: Short-term memory
UN: United Nations
